# Chromosomal coordination and differential structure of asynchronous replicating regions

**DOI:** 10.1038/s41467-021-21348-4

**Published:** 2021-02-15

**Authors:** Britny Blumenfeld, Hagit Masika, Marganit Farago, Yishai Yehuda, Lamia Halaseh, Oriya Vardi, Rachel Rapoport, Rena Levin-Klein, Howard Cedar, Yehudit Bergman, Itamar Simon

**Affiliations:** 1 Department of Microbiology and Molecular Genetics, IMRIC, Hebrew University Medical School, Jerusalem, Israel; 2 Department of Developmental Biology and Cancer Research, Hebrew University Medical School, Jerusalem, Israel; 3 Department of Bioinformatics, Jerusalem College of Technology, Jerusalem, Israel

**Keywords:** DNA replication, Epigenetics

## Abstract

Stochastic asynchronous replication timing (AS-RT) is a phenomenon in which the time of replication of each allele is different, and the identity of the early allele varies between cells. By taking advantage of stable clonal pre-B cell populations derived from C57BL6/Castaneous mice, we have mapped the genome-wide AS-RT loci, independently of genetic differences. These regions are characterized by differential chromatin accessibility, mono-allelic expression and include new gene families involved in specifying cell identity. By combining population level mapping with single cell FISH, our data reveal the existence of a novel regulatory program that coordinates a fixed relationship between AS-RT regions on any given chromosome, with some loci set to replicate in a parallel and others set in the anti-parallel orientation. Our results show that AS-RT is a highly regulated epigenetic mark established during early embryogenesis that may be used for facilitating the programming of mono-allelic choice throughout development.

## Abstract (web summary)

Most regions of the mammalian genome replicate both alleles in a synchronous manner, but some loci have been found to replicate asynchronously and the time of replication of each allele is different. Here the authors, by employing clonal mouse cells from a hybrid strain chart replication timing over the entire genome, using polymorphisms to distinguish between the paternal and maternal alleles.

## Introduction

The entire genome is divided into distinct ~1–2 Mb zones that replicate in an ordered and coordinated manner during S phase. Early replicating domains co-map with light chromosome G-bands, have a relatively open DNaseI-sensitive chromatin structure and carry many expressed genes. In contrast, late replicating domains are embedded within dark G-bands, are structurally less accessible and seem to house genes that are not normally transcribed^1^. In many cases, the activation of tissue-specific genes during development is accompanied by a local programmed change to early replication^2^, suggesting that there is a direct cause-and-effect connection between replication timing and gene expression and experiments in vitro provide some support for this idea^3,4^.

While most regions of the genome replicate both alleles in a synchronous manner, some loci have been found to replicate asynchronously, with one allele undergoing DNA synthesis early in S phase and the other replicating during late S^5–7^. This pattern is typical of imprinted gene regions, which are evidently set up during gametogenesis to replicate early on the paternal allele and late on the maternal, a pattern that is maintained during development and is then present in all somatic cell types^8^. A second type of asynchronous replication timing (AS-RT) pattern is characterized by cell heterogeneity with some having the maternal allele early replicating while in others, the orientation is reversed, with the paternal allele being early^7,9^. In this case, as well, the asynchronous (AS) replicating mode is set up early in development and is then preserved in all somatic cell types where the maternal/paternal orientation is often maintained in a clonal manner^6^. Many known genes and gene arrays are located in these AS-replicating domains, including those from the immune and olfactory systems, where this property is associated with monoallelic expression^9^. Indeed, in the case of the Igκ and Igμ gene arrays, it has even been shown that it is always the early replicating allele that is initially chosen for expression^7^.

AS-RT was first uncovered using FISH technology to visualize the generation of DNA strands at individual sites in the genome^10,11^. When assayed in S-phase nuclei, each locus can be visualized as a single dot prior to being copied, but as two hybridization dots following replication. Thus, for any given probe, the time of replication can be deduced from the number of nuclei having double dots. For most loci, the two alleles behave in a synchronous manner so that nuclei have either two single dots (one for each allele) or two double dots. The observation of many nuclei with one single and one double dot therefore indicates that one allele has already replicated while the other has not, suggesting AS replication. In a few cases, this allelic difference in replication timing has been confirmed by S-phase fractionation of BrdU-labelled DNA^6–8^.

Since this technology can only be applied at individual loci, it has not yet been possible to actually map all the AS regions in the genome. While several studies have employed genome-wide approaches for mapping replication timing, AS regions always remained undetected, since some cells replicate the maternal allele early while in others it is the paternal allele that is copied early, thus cancelling out these differences in a population of cells. In this paper, we solved this problem by employing clonal cells from a hybrid cross between C57BL6 (B6) and Castaneous (Cast) mice to chart replication timing over the entire genome, using polymorphisms to distinguish between the paternal and maternal alleles. These experiments have revealed hundreds of previously undiscovered asynchronously replicating sites, allowing us to characterize these regions, gain insight into the regulation of this phenomenon and decipher the structure-function relationship between replication timing and gene expression.

## Results

**Fig. 1:**
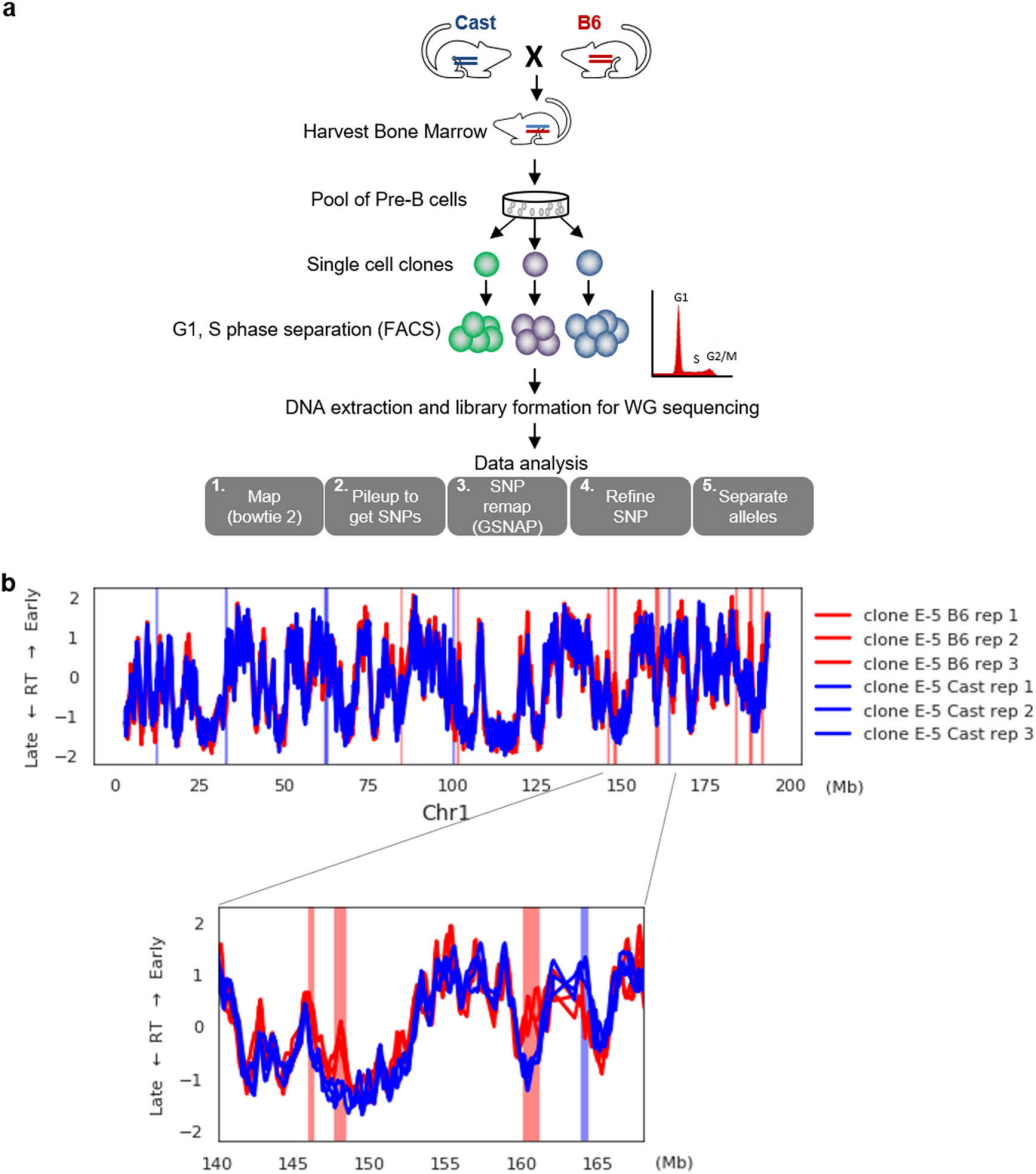
Whole-genome allele-specific replication-timing analysis. **a** Experimental design. **b** Replication timing (RT) of Cast (blue) and B6 (red) alleles (in clone E-5) on chromosome (Chr 1) for three individual replicates, with a magnified view of an ~25 Mb region. Relative replication time in S phase is on a scale from −2 (very late) to +2 (very early). Asynchronous regions are indicated by a red (B6 early) or blue (Cast early) stripe.

Although a number of studies have attempted to map DNA replication patterns on a genome-wide basis^12–15^, it has not been possible to detect all regions that replicate asynchronously, especially in those cases where there is a random distribution, with some cells replicating the paternal allele early while in others it replicates late. These regions can be identified at individual sites using a FISH assay, but it is hard to detect them using high throughput technology because, having both directions within the cell population, cancels out the differential replication-timing pattern. In order to overcome this problem, we generated F1 animals from a cross between B6 and Cast mice and isolated individual bone-marrow-derived pre-B cell clones for replication-time analysis (Fig. 1a). Since these strains can be distinguished by nearly 15 million SNPs^16^, it was possible to measure the replication time of each allele separately over almost the entire genome. Previous studies have shown that AS-replication-time patterns present in somatic cells are maintained in a clonal manner, thus insuring that all cells behave with the same replication-time properties^6,7^.

We used DNA-content^17,18^ for mapping the genome-wide replication-time (RT) profile in a single pre-B cell clone (E-5). We first identified SNPs by mapping sequencing results to the mm9 genome and these were then employed to assign the reads to alleles by remapping each read with GSNAP^19^ using the predetermined SNP file. With this methodology, we avoided reference mapping bias (see Methods). RT was mapped in triplicate from independent experiments and we then used the likelihood-ratio-test method to identify all genomic regions that show significant differences in RT between the two alleles. These results revealed 194 autosomal AS regions, with some of them showing earlier replication on the Cast allele while others had a reverse pattern with this allele replicating late in the cycle (Fig. 1b). Comparing these patterns to those seen in pure B6 or Cast cell lines, we noted that a number of AS sites were due to a genetic difference between the strains (Supplementary Fig. 1a). After removing these loci, we were left with 125 epigenetic AS regions. Since this experiment revealed many regions of the genome not previously identified as asynchronously replicating, we first tried to verify these sites by using an alternate assay. To this end, we carried out FISH analysis to measure single/double hybridization dots on 16 separate loci that showed differential replication by whole-genome analysis (Table 1). For all of these selected regions, a high percentage (>35%) of interphase nuclei were characterized by a single/double pattern, signifying that one allele replicates prior to the other. In contrast, synchronously replicating control regions showed this pattern in <23% of the nuclei, within the range of background level.

**Table 1:** Asynchronous replication timing by FISH analysis.

		SD (%)					SD (%)	
Chr	Bac name	Clone E-5	Clone C2	Clone B4	ES (V6.5)	MEF	YC	hChr
3	RP23-341D14	56 (*n* = 57)	58 (*n* = 31)	—	—	—		
3	RP23-235F22	45 (*n* = 95)	—	—	—	—		
3	RP23-317D10	51 (*n* = 60)	42 (*n* = 87)	—	50 (*n* = 109)	—		
3	RP23-115D3	42 (*n* = 90)	42 (*n* = 88)	—	43 (*n* = 108)	—		
4	RP23-439A9	54 (*n* = 55)	51 (*n* = 76)	38 (*n* = 96)	50 (*n* = 100)	40 (*n* = 102)	38 (*n* = 76)	6
6	RP24-100L23	59 (*n* = 117)	39 (*n* = 104)	47 (*n* = 104)	57 (*n* = 116)	58 (*n* = 68)		
8	RP24-318I18	58 (*n* = 78)	56 (*n* = 79)	40 (*n* = 87)	35 (*n* = 101)	52 (*n* = 106)		
4	RP24-83L7	52 (*n* = 96)	58 (*n* = 52)	—	45 (*n* = 103)	48 (*n* = 108)	50 (*n* = 103)	8
8	RP23-182L17	38 (*n* = 32)	—	41 (*n* = 92)	44 (*n* = 110)	47 (*n* = 103)		
16	RP23-326K16	53 (*n* = 76)	—	47 (*n* = 74)	42 (*n* = 96)	61 (*n* = 96)	50 (*n* = 79)	21
16	RP23-38D22	53 (*n* = 43)	57 (*n* = 65)	59 (*n* = 105)	50 (*n* = 112)	57 (*n* = 104)	44 (*n* = 89)	21
12	RP24-386G17	—	54 (*n* = 56)	47 (*n* = 103)	47 (*n* = 96)	47 (*n* = 101)		
12	RP23-13F5	46 (*n* = 103)	43 (*n* = 91)	47 (*n* = 105)	52 (*n* = 109)	52 (*n* = 65)		
12	RP23-333P23	52 (*n* = 57)	39 (*n* = 84)	—	—	—		
4	RP23-63H2	55 (*n* = 88)	—	—	—	—		
16	RP23-59I11	50 (*n* = 83)	—	—	—	—		
**6**	**RP23-68K13**	**23** (*n* = 81)	**10** (*n* = 109)	**18** (*n* = 100)	**12** (*n* = 108)	**21** (*n* = 95)	**23** (*n* = 104)	**7**
**13**	**RP24-353L13**	**15** (*n* = 100)	**11** (*n* = 100)	**13** (*n* = 97)	**18** (*n* = 101)	**10** (*n* = 106)		
**15**	**RP23-81A7**	**17** (*n* = 101)	**20** (*n* = 102)	**12**(*n* = 101)	**17** (*n* = 100)	**10** (*n* = 106)		

FISH was carried out in five mouse cell populations, hybrid clones E-5 and C2, C57BL/6 pre-B cell clone B4, ES cells-, and MEFs, as well as on human lymphoblasts (YC) using different BAC probes representing regions determined to be asynchronously or synchronously (bold type) replicating by whole-genome analysis. The *p* value for the difference between asynchronous and synchronous replication was 0.008 as determined using the one-sided Mann–Whitney test. The percentage of nuclei with a single/double pattern is recorded. Note that the human cells were assayed using probes syntenic to the corresponding mouse region. The human chromosomal location of these probes is also listed (hChr).

**Fig. 2:**
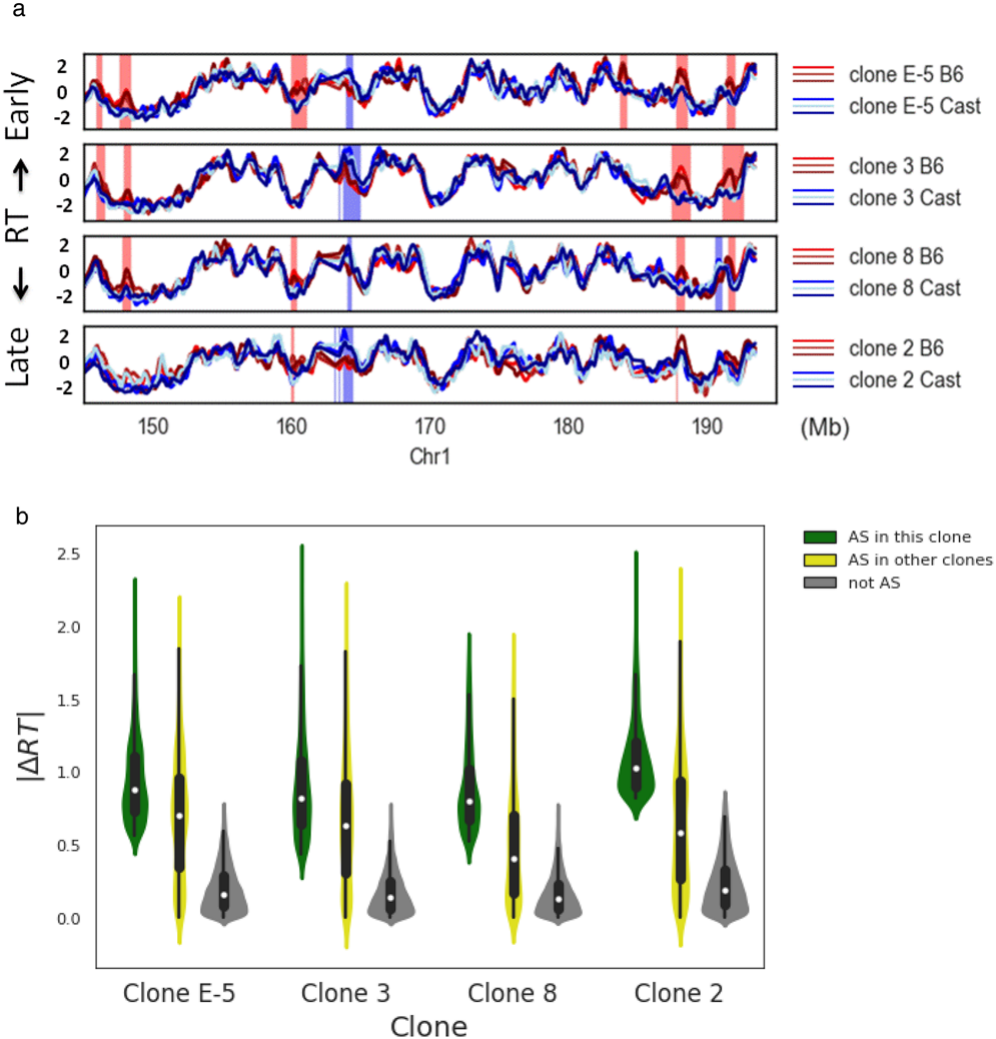
Comparison of AS-RT in pre-B cell clones. **a** Replication timing (RT) of the B6 (red) and Cast (blue) alleles (three replicates) on Chr1 for different pre-B-cell clones. Asynchronous regions detected in each clone are marked by red (B6 early) or blue (Cast early) stripes. **b** Violin plots showing allelic replication-timing differences (|∆RT|) for both the B6 and Cast early regions on all chromosomes for a given clone (green). This is compared to |∆RT| in the same clone for asynchronous detected only in other clones (yellow) or for regions of the genome that were never detected as being asynchronous (grey). Each box shows the quartiles of the dataset while the whiskers extend to show the rest of the distribution, except for points that are determined to be outliers (>upper quartile +Iqr*1.5 or <lower quartile −Iqr*1.5). The number (n) of points included in each box ranged from 900–2000 for the AS loci and 13,000–22,000 for the non AS loci.

We next used this whole-genome approach to ask whether this same asynchronously replicating pattern is observed in other independently-isolated pre-B cell clones, as well. Results from three additional clones show extensive overlap between the AS regions detected in E-5 and the newly analyzed clones (Fig. 2a and b). While not all loci asynchronously replicating in one clone were necessarily found to be asynchronously replicating in the others (Supplementary Fig. 1b, c), quantitative analysis indicated that most sites detected in our assay showed some degree of differential replication timing between the alleles in other clones as well, although this difference did not always reach the threshold level required to be scored in the whole-genome assay (Fig. 2b). It thus appears that while there is some variability between clones, all 326 unique regions definitively identified by this method (Supplementary Table 1) demonstrate AS-replication properties in almost every clone tested. Furthermore, using FISH methodology, we could demonstrate that these regions have a significantly higher percentage (*p* = 0.008) of nuclei with a single/double pattern in all of the tested clones (Table 1), even when the genome-based method only revealed weak differential replication timing. Similar results were obtained using normal nonhybrid pre-B cells (B4), as well. Logarithmic regression analysis (using the nls R function) of our data (Supplementary Fig. 1c) indicated that, in total, there are no less than ~500 AS-replicating domains in the genome.

All AS-replicating regions found until now, regardless of the cell type in which they were originally identified, demonstrated this same pattern in other somatic cell types as well as in embryonic stem cells (ES). In order to determine whether the regions detected in our genome-wide assay are also AS-replicating in other cell types, we used FISH to examine both ES cells and MEFs at a number of different sites (*n* = 11) located on a variety of chromosomes (Table 1). This analysis indicated that these regions behave similarly to standard AS-replicating markers. Furthermore, this phenomenon was also detected in human syntenic chromosomal regions chosen at random (Table 1). In each case, the number of nuclei showing a single/double hybridization pattern was significantly (*p* < 10^−4^ Wilcoxon rank-sum test) different than the synchronously replicating control. While these data clearly support the idea that AS-RT is set up very early in development^7,8,20^, the mechanism for this is not understood, although there is evidence that the histone modification pattern at replication origins may play a role^21,22^.

It has recently been shown that it is possible to measure whole-genome replication timing in single cells^23,24^. Although these authors did not attempt to identify random AS-replication regions, we have taken advantage of a subset of their data obtained from two-strain hybrid cells^23^ to mine information on this phenomenon by scoring S-phase cells for the presence of one replicated and one unreplicated allele in a manner similar to single/double dots in the FISH assay. In this manner we were able to show that the set of AS-replicating regions mapped in our experiments is enriched (*p* < 10^−66^; Chi square test) for asynchrony in their data (Supplementary Fig. 2a), with some replicating the paternal allele early and others, the maternal (Supplementary Fig. 2b). These data thus serve to validate our genome mapping results in a nonclonal population obtained from a completely different cell type.

### Characterization and expression of AS-RT regions

As a first step in characterizing the newly found AS regions, we tested them for known genomic features. In general, they were found to replicate relatively late in S phase (Supplementary Fig. 2c), have relatively high long interspersed nuclear element (LINE) and low short interspersed nuclear element (SINE) densities (Supplementary Fig. 2d). These features are characteristic of late replication-time domains^25,26^. Further analysis revealed that these regions are also associated with late replication-time epigenetic features such as enrichment for the B compartment^27^, lamin associated domains (LADs) and lower gene expression levels (Supplementary Fig. 2).

**Fig. 3:**
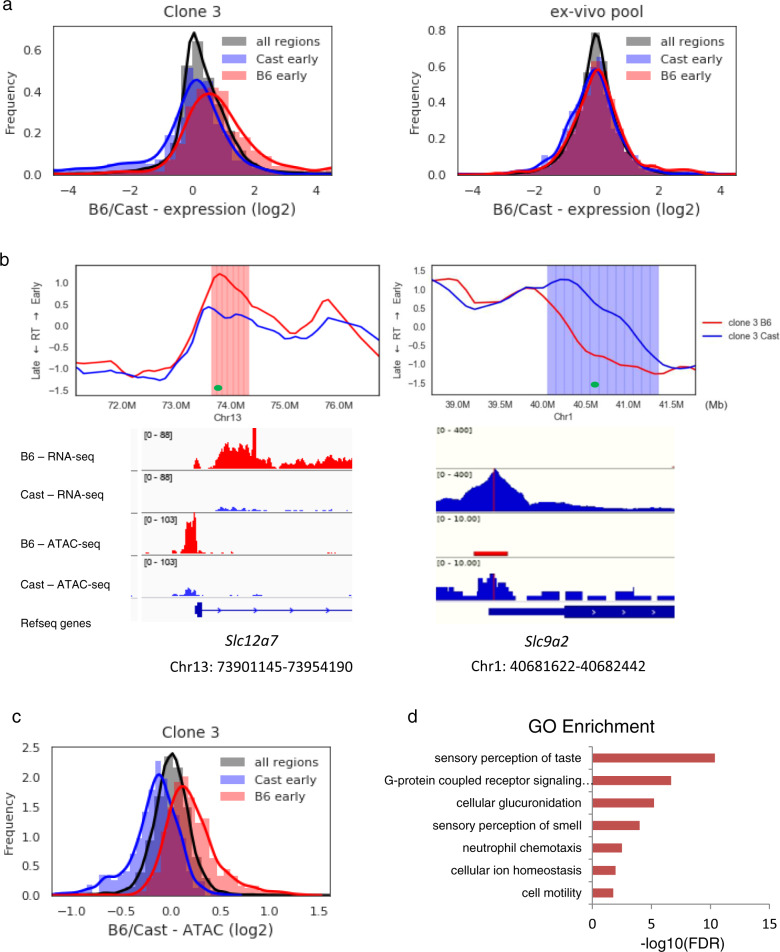
Characterization of AS-replicating regions. **a** Distribution plot for expression ratio of Cast early (blue) and B6 early (red) regions (p for difference <10^−45^; one-sided *t*-test) based on allele-specific RNA-Seq data from clone C3 (left) or from an ex-vivo pool of pre-B cells (right). **b** IGV track depicting gene-specific RNA-Seq coverage and ATAC profile for the B6 (red) and Cast (blue) alleles from two different asynchronously replicating regions in clone C3. The AS-RT replication patterns are shown above each example. *Slc12a7* is in a B6 early region, while *Slc9a2* is located in a Cast early replicating region, as determined by whole-genome analysis. **c** Distribution plot of ATAC-seq ratios (*p* < 10^−98^; one-sided *t*-test). **d** Gene ontology bar plot of asynchronously replicating regions.

Previous studies have demonstrated that AS-replicating regions may be associated with specific gene sets that are expressed monoallelically, such as the olfactory receptor genes in nasal epithelium or immunoglobulin genes in the immune system^5,9,28^. On this basis, we next asked whether these genomic asynchronously replicating regions are also enriched for monoallelic transcription units. To this end, we analyzed RNA-Seq data from B6/Cast pre-B cell clones^29^ and scored the presence of all transcripts measured from each allele. As shown in histogram form (Fig. 3a), as opposed to most regions in the genome, expression in these AS domains is highly skewed to one allele over the other and this can be observed in many specific examples, as well (Fig. 3b and Supplementary Fig. 3a).

Regions in which the Cast allele replicates earlier than the B6 allele, are transcribed preferentially (*p* < 10^−45^; *t*-test) from the Cast allele and the opposite was true for regions in which the B6 allele replicates at the earlier time, even though many of the RNAs do not represent actual gene transcripts. As expected, skewing was not observed where RNA-Seq was carried out on an ex-vivo B6/Cast cell pool (Fig. 3a). It is likely that this allele-specific expression is due to differential chromatin structure. Indeed, using ATAC-Seq to profile accessibility, our analysis indicated that the early expressed allele is in a more open configuration than the corresponding late allele (*p* < 10^−98^; *t*-test) in all cases (Fig. 3c). Interestingly, gene-specific differential ATAC peaks were observed within AS-replicating regions, even when the specific gene was not expressed (Supplementary Fig. 3b), suggesting that these regions maintain structural signatures independent of transcription.

Based on a small number of previously identified AS-replicating regions in the genome, it appeared that these regions are enriched for genes involved in cell interactions with its outside environment, such as olfactory receptors, immunoglobulins and other receptors located in the immune system^5^. Having a genome-wide map of AS regions, however, now allowed us to examine this question in a more general manner. Using a gene ontology (GO) search to characterize these regions, additional gene categories including taste (gustatory) receptors, chemotaxis and cell motility were identified (Fig. 3d). Furthermore, analysis of RNA-Seq data from pre-B cell clones indicates that many of these genes are indeed expressed monoallelically (Fig. 3b and Supplementary Figs. 3a, c), suggesting that AS-RT may actually play a role in this process.

### Chromosomal coordination of AS-RT regions

**Fig. 4:**
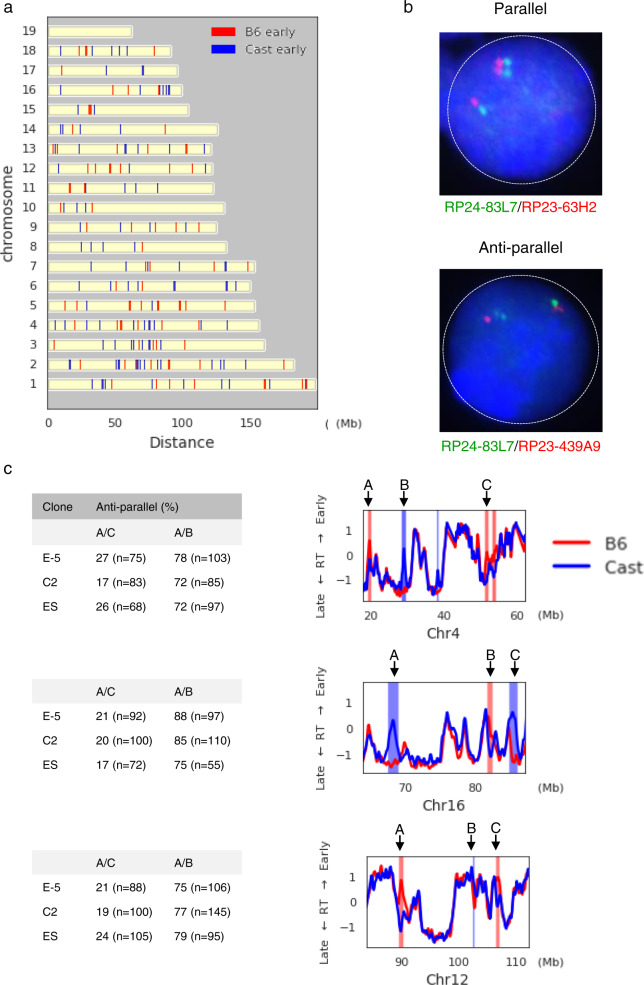
Orientation of asynchronous replication regions. **a** Whole-genome chromosome map of all AS-replication regions’ orientation (Cast early, blue; B6 early, red). **b** Representative double-label FISH images showing nuclei with single/double hybridization patterns for two probes on the same chromosome. Probes R-83L7 and RP23-63H2 show a parallel pattern of hybridization with both double signals on the same allele (top), while probes RP24-83L7 and RP23-439A9 show an antiparallel pattern with each allele carrying one single and one double hybridization signal (bottom). **c** Comparison of asynchronous region pairs (A/B or A/C) on three different chromosomes (Chr16, Chr4, Chr12) using double-label FISH as shown in **b**. Table shows the percent of single/double nuclei with antiparallel allelic replication patterns. The position of regions A, B, and C for each chromosome is shown on the adjacent replication-time (RT) maps (right) derived from clone E-5. All measurements were carried out on two independent pre-B cell clones and in ES cells. The maximum *p* value for all cases was <2 × 10^−4^ as determined by the two-tailed binomial test. BAC probes used in this experiment were as follows: Chr16 (A = RP23-38D22, B = RP23-326K16, C = RP23-59I11); Chr4 (A = RP24-83L7, B = RP23-439A9, C = RP23-63H2); Chr12 (A = RP23-13F5, B = RP24-386G17, C = RP23-333P23). *n* = number of cells counted in each clone.

Several studies have attempted to determine the relationship between asynchronously replicating regions on the same chromosome. FISH analysis of select probes suggested that the majority of AS loci on any given chromosome always replicate in the same orientation^30,31^, with only a few exceptional sites having an opposite pattern^32,33^. The identification of many new AS-replicating loci by our genome-wide analysis (Fig. 2), however, has now made it possible to obtain a comprehensive site-orientation map for every chromosome in the cell (Fig. 4a). In contrast to what was expected from previous studies, it now appears that almost all chromosomes have some loci in one orientation, with early replication on the maternal allele and a relatively equal number of other loci replicate in the opposite orientation, with the early allele being on the paternal copy. This pattern could have easily been missed in previous studies that examined only a small number of loci (*n* = 2–4) on each individual chromosome^29,30^.

In order to verify this pattern of parallel and antiparallel asynchronous replication orientation (see below), we adopted a pairwise FISH strategy to examine individual loci in the same pre-B cell clones that had been subject to whole-genome analysis (Fig. 2). To this end, we first carried out FISH on nuclei from clone E-5 using individual fluorescent probes for two different loci on a single chromosome and then identified cells exhibiting a single/double pattern for both sites. If both loci have the single (late replicating) signal on the same chromosome, this indicates that these loci replicate in the same orientation, both undergoing early replication on the same allele (parallel coordination). In contrast, if nuclei have the opposite pattern, with the single signal of one locus being on the same allele as the double signal of the other locus, this suggests that the two asynchronously replicating sites do so in an opposite orientation (antiparallel coordination) (Fig. 4b).

We first selected three specific regions on Chr4 that had been identified by genome-wide analysis of clone E-5 (Fig. 4c). Regions A and C were found to undergo asynchronous replication in a parallel orientation. In keeping with this, FISH analysis using probes for these regions (RP23-63H2 and RP24-83L7) showed a parallel pattern (Fig. 4c). In contrast, pairwise analysis of regions A and B on this same chromosome showed an antiparallel pattern, consistent with the observation that these two regions undergo antiparallel replication timing. Similar results (Fig. 4c and Supplementary Fig. 4) were obtained using probe-sets representing parallel and antiparallel regions on other chromosomes (3, 8, 12, and 16). Taken together, these findings yielded results completely consistent with whole-genome replication-timing analysis showing that both parallel and antiparallel asynchronous replication exists on all tested chromosomes (Fig. 4 and Supplementary Fig. 4).

Previous studies have shown that most AS-replicating regions can be early replicating on the maternal allele in some cells, but on the paternal allele in others and it has been demonstrated that these epigenetic states can switch from one to the other in both embryonic and adult stem cells^20^. In contrast, the AS-replication orientation of each locus appears to remain fixed in individual clonal populations of somatic cells, and it is indeed this property that allowed us to measure genome-wide AS replication. Surprisingly, we obtained very similar locus orientation maps in independent pre-B cell clones isolated from B6/Cast mouse bone marrow (Fig. 2a and Fig. 4c). It is very likely that this resulted from growth selection occurring during the process of cloning hybrid cells in vitro. As compared to B6, the Cast genome has many deletions and some of them are very large^16^. Thus, a monoallelic pattern in these regions could clearly generate cells that are completely lacking the expression of important genes, thus bringing about selection for particular replication-timing directions and elimination of others. A similar phenomenon is associated with X inactivation in females carrying deletions or mutations on this chromosome^34^.

**Fig. 5:**
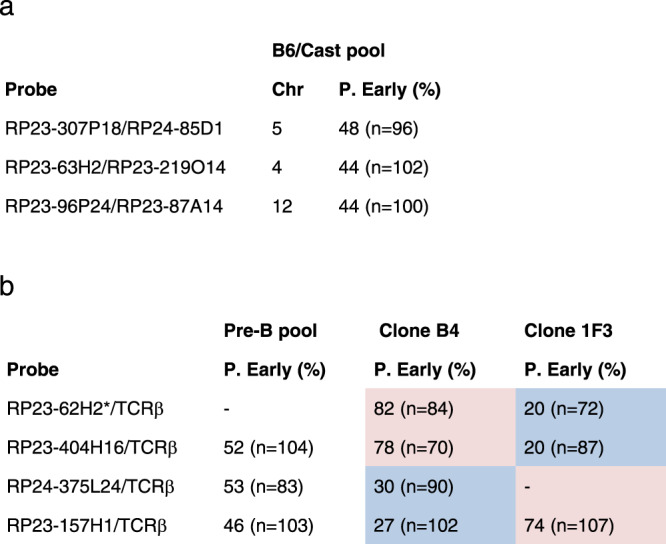
Asynchronous replication orientation. Double-label FISH analysis was carried out on a pool of B6/Cast pre-B cells (**a**), as well as a pool of pure B6 pre-B cells carrying a large *Tcrβ* deletion on the paternal allele of Chr6 and two individual clones (B4 and 1F3) isolated from this population (**b**). In each FISH experiment, one fluorescent probe (listed first) was specific for an asynchronous replicating region, while the second probe (listed second) was used to specifically detect the nondeleted maternal allele using either the *Tcrβ* probe on Chr6 or probes specific for regions naturally deleted on the Cast allele (paternal). Nuclei with single/double signals were selected and counted to determine the percentage having an early paternal allele (double). The *p* value for pools was >0.1 and for specific clones, <10^−4^ as determined by the two-tailed binomial test. Paternal early (red background) or maternal early (blue background) are marked. The first two probes and the last two probes are located in regions determined by whole-genome analysis to be antiparallel. (*) Note that this is the probe for the olfactory receptor region on Chr6.

To reaffirm that for any given locus some cells replicate the maternal allele early while in others it is the paternal allele that replicates early, we isolated a bone-marrow-derived pool of B6/Cast pre-B cells and measured replication timing of select loci using two-color FISH, with one probe detecting the AS locus and the other, specific for deletions present in the Cast genome, used for identifying the parental allele^6,20^. This experiment showed that for each locus, some cells have the paternal allele early, while in others it is the maternal, clearly indicating that both orientations exist in this mixed population (Fig. 5a). Similar results were obtained using a pool of pure B6 pre-B cells (marked with a deletion in the *Tcrβ* locus on Chr6) (Fig. 5b). We then succeeded in isolating single-cell-derived clones from this pure B6 population (carrying nonbiased identical alleles) and were able to identify two individual clones that demonstrate an opposite orientation for each probe tested on chromosome 6 (Fig. 5b), consistent with the genomic analysis of these sites. This concept was also confirmed independently using data mined from single-cell replication-time analysis in hybrid strains^23^ showing that any given allele replicates early in some cells and late in others (Supplementary Fig. 2b).

**Fig. 6:**
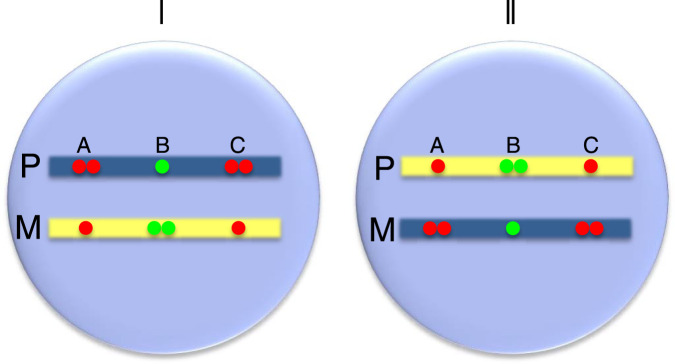
Orientation of asynchronous regions. All cells are in one of two states (I or II), Paternal (P, blue), Maternal (M, yellow). Probes A and C replicate in a parallel manner, with both being early on the same allele, while probes A and B always replicate in an antiparallel manner both in cell I and cell II.

These experiments, involving multiple parallel and antiparallel probes in two complementary clonal populations, strongly suggest that there may actually be a fixed relationship between AS loci on any given chromosome. To verify that this indeed is the case, we carried out pairwise FISH analysis to determine the relative orientation of multiple probes on a given chromosome in a number of different nonclonal cell populations, including fibroblasts (MEF), T lymphocytes and ES cells (Fig. 4c and Supplementary Fig. 4). In all cases, the orientation relationship between multiple probes on three different chromosomes was identical to that seen in the original pre-B cell clones (Fig. 4). Strikingly, we were also able to confirm this general rule by demonstrating that two individual probes located within a syntenic region on Chr 21 in human lymphoblast cells maintain this exact same pattern (Supplementary Fig. 4c). It should be noted that all of these examples represent a mixture of cells with opposite orientation at each AS site. Thus, even though the orientation of each individual locus is not identical in every cell, its relationship to other loci on the same chromosome is fixed, being either parallel or antiparallel (Fig. 6).

It was previously shown that gene families from the immune (e.g., immunoglobulins and interleukins) and olfactory system are asynchronously replicating in human cells in a manner similar to those in the mouse genome^30^. Our studies (Table 1) now indicate that this replication-time homology may extend to many additional syntenic regions of the human genome. Indeed, every tested homologue of AS loci identified by our genome-wide replication assay in mice was shown to replicate asynchronously in human cells, as well. Furthermore, in at least one case, we demonstrated that two loci with an antiparallel orientation pattern in mouse, retains this same orientation in its human syntenic region, as well (Supplementary Fig. 4c). This suggests that there may be a common program for setting up the AS-RT landscape in both organisms.

## Discussion

A small number of random AS regions have been previously detected by FISH analysis of interphase nuclei from S-phase cells where one can actually visualize local chromosome replication by counting the number of alleles detected by hybridization to specific probes. In this paper, we have taken advantage of hybrid B6/Cast mice to map the genome-wide random AS-RT landscape in pre-B cell clones. Using strict criteria to detect asynchrony, we were able to identify many new regions, thus greatly expanding and enriching our knowledge of this phenomenon. It has been noted that the FISH assay is dependent not only on the timing of DNA synthesis, but also on visual segregation of the newly replicated alleles. Indeed, there have been several reports on individual loci that show only minimal differences in the actual time of DNA synthesis despite a clear-cut asynchronous FISH pattern^35–38^. In addition, there is a question of the significance of AS replication in terms of understanding how this might impinge on chromatin structure and function.

Our results have helped resolve some of these issues. By taking into consideration the full complement of AS regions identified by whole-genome analysis, it appears that the degree of differential DNA synthesis timing between the alleles is somewhat variable between clones. Nonetheless, they are always found associated with a profile of differential chromatid segregation, as revealed by FISH analysis and this structural manifestation is seen in all cell types tested. It should be noted that if the degree of differential DNA synthesis timing is low, it may not register as being above the threshold for detection by low-resolution cell cycle assays. This may explain why AS-replicating domains previously identified by FISH (e.g. olfactory and immune system receptor regions, as well as imprinted loci) were not necessarily detected by our^5^ or previous^12,13^ whole-genome analyses and only registered small differences in standard cell-fractionation based DNA replication-timing assays^7,8^. The fact that differential segregation timing between the two alleles (FISH) is a major component of this phenomenon, strongly suggests that there must be underlying structural differences between the two alleles. Our studies support this idea by showing that there is indeed a clear-cut global difference in allele accessibility at these loci. In light of these findings, we suggest using the term “Random Asynchronous Chromosome Replication Timing” (RACRT) to describe this phenomenon.

In an attempt to understand the functional significance of these domains, we integrated RNA-Seq on the B6/Cast clones analyzed in this paper, using the many polymorphic differences between the two strains in order to identify the allelic source of each transcript. Expression of transcripts within the AS domains was highly skewed to the early replicating allele, in keeping with the finding that this same allele is also in a more open chromatin configuration (ATAC). This is consistent with previous work on individual loci^6,7,13,39^. It should be noted that the pattern of AS replication is set up very early during embryogenesis^7,40^ and is then maintained in all cell types of the organism. This suggests that allele-specific structures are initially generated by recognition of their target loci in a manner that is independent of RNA transcription (Supplementary Fig. 3b). Thus, RACRT represents a sophisticated general genome-wide mechanism to dictate monoallelic expression in many different somatic cell types on the basis of preset differential structural features that determine which allele is preferentially targeted by the transcription machinery^41–43^. Thus, while the general relationship between early RT and gene expression is complicated by the fact that other parameters may contribute to transcription regulation, in the case of AS-RT, this cis acting mechanism probably plays a more dominant role, since both alleles are equally exposed to transcription factors. Nonetheless, there is evidence that there may also be trans-acting mechanisms for generating sites of monoallelic expression at other loci^43^.

These results add to the growing list of findings suggesting that RACRT plays a causal role in allelic choice. First, evidence in vivo^7,40^, as well as in ES cells^20^ has shown definitively that this allele-differential mark is established very early in development, prior to the appearance of monoallelic expression in somatic cells. Furthermore, it appears that differential chromatin accessibility is also set up independently of expression, as indicated by the observation that it is present even in cell types that do not have active transcription at these loci (Supplementary Fig. 3). Finally, it should be noted that direct nuclear injection experiments demonstrate that DNA undergoing replication in early S phase is differentially packaged into open chromatin and expressed, as opposed to the inactive state observed for this exact same template when it undergoes replication in late S^3,4^. While these experiments may not constitute formal mechanistic proof for a causal role of RACRT in the generation of monoallelic expression, it certainly provides very strong support for this idea.

AS-replicating regions of the genome, as detected by FISH, have been shown to harbor a variety of gene arrays mostly coding for receptor-like proteins that are used for cell–cell or cell–environment interactions, such as those associated with the immune system, the olfactory system and a variety of cytokine-like genes^5^. Genome-wide bioinformatics analysis has allowed us to expand this list by highlighting the gustatory system as well as genes involved in chemotaxis and cell motility. Indeed, examination of expression patterns in our pre-B-cell clones indicate that chemotaxis genes may also be transcribed monoallelically (Supplementary Fig. 3c). These findings provide additional support for the idea that AS-replication regions are part of a mechanism for generating cell-interaction diversity by bringing about monoallelic expression in individual cells^41,44^.

With the physical mapping of genome-wide AS sites, it has now become possible to investigate the regulatory rules that govern this process. Our results have already answered one of the key questions by clearly demonstrating that in any given cell, each chromosome carries sites that replicate with the paternal allele early together with sites that replicate in the opposite direction, with the maternal allele early. Using a pairwise FISH assay to examine separate loci on a single chromosome, we have shown that there is a fixed relationship between any two independent pairs, replicating either in parallel or in an opposite orientation to each other. This same highly coordinated pattern is seen in the clones we have examined, but is also observed in pools of pre-B cells (which include both orientations for each site) and even in ES cells, which are characterized by constant switching from cell generation to generation^20^.

Thus, there appears to be a fixed orientation map of AS replication on each chromosome, with some loci set to replicate in a parallel orientation and others set in the antiparallel direction (see Fig. 6). While this structural plan appears to be true for all individual chromosomes, the FISH assay is not easily amenable for determining the relationship between loci on different chromosomes. On one hand, it may be that the overall orientation on each individual chromosome is set up independently, thus producing a large variety of cells carrying different interchromosomal mixtures. Alternatively, there may actually be a fixed relationship between all AS regions in the genome, perhaps being set up this way at the very beginning of embryogenesis on the basis of parentally derived signals. Our results showing that every pre-B cell clone had exactly the same overall AS-replication orientation map strongly supports (see Methods) the idea of a fixed genome-wide program. If this is indeed the case, every cell in the body would carry only one of two possible AS-replication patterns with some cells having the precise orientation map of the pre-B clone used in this study and others having the exact opposite antiparallel orientation at every AS locus.

Taken together, our studies reveal a highly coordinated program for marking multiple regions of the genome in an allele-differential manner and these encompass many important gene functions that help define cell identity, thus generating single-cell diversity. The entire system is built on one of the most fundamental components of eukaryotic cell biology, the timing of chromosome replication, which appears to represent an autonomous layer of epigenetic regulation. Once established in the early embryo, it is then maintained in all future cell descendants where, by affecting regional genome accessibility, it provides a template for directing allele-specific choice. Significantly, our findings show that homologous regions in the human genome also undergo RACRT and are subject to an identical pattern of programmed coordination, suggesting that this process may be ubiquitous in nature and, as such, probably plays an important role in biology.

## Methods

### Mice/animals

C57BL/6 (B6) mice (Harlan) were crossed with wild-type M. Castaneous (Cast) mice (Jackson Laboratories) to generate B6/Cast hybrid mice. Mice were housed and cared for under specific pathogen-free conditions. We have complied with all relevant ethical regulations for animal testing and research as approved by the Animal Care and Use Committee of the Hebrew University of Jerusalem.

### Cells and cultures

To prepare pre-B-cell clones, cells isolated from mouse bone marrow (10 weeks old) were grown in RPMI 1640 media (Gibco) supplemented with 10% fetal bovine serum (HyClone), 100 U/ml penicillin, 100 μg/ml streptomycin (Gibco), L-glutamine (Gibco), and 50 μM of β-mercaptoethanol (Gibco) on irradiated ST2 feeder cells (provided by A. Rolink). Recombinant mouse IL-7 (10 μg/ml) was added to select for pre-B cell populations (Peprotech, USA). After 10–14 days of IL-7-mediated positive selection, cells were plated on 96-well plates in limiting dilutions to generate single-cell-derived pre-B cell clones. Embryonic stem (ES) cells (C57BL/6) were cultured without feeders on 0.2% gelatin-coated plates in DMEM supplemented with 15% inactivated fetal calf serum (HyClone), 1 mM L-glutamine, 100 U/ml penicillin, 100 μg/mL streptomycin, 0.1 mM β-mercaptoethanol, 1 × nonessential amino acids and homemade leukemia inhibiting factor (LIF). Mouse (C57BL/6) embryonic fibroblasts (MEFs) were grown on DMEM supplemented with 15% inactivated fetal calf serum (HyClone), 1 mM L-glutamine, 100 U/ml penicillin, 100 μg/ml streptomycin, 0.1 mM β-mercaptoethanol. Human lymphoblast cells (YC) and mouse T cells were grown in RPMI 1640 media (Gibco) supplemented with 10% fetal bovine serum (HyClone), 100 U/ml penicillin, 100 μg/ml streptomycin, L-glutamine (Gibco), and 50 μM of β-mercaptoethanol (Gibco).

### Flow cytometry (FACS)

Cells were prepared and sorted as previously described^45^. In brief, cells were gently washed twice with ice-cold PBS and resuspended in 250 µl cold PBS. For all cells, 95% high purity EtOH was added dropwise while slowly vortexing to a final volume of 70% EtOH and then incubated for 24 h at 4 °C. Fixed cells were washed once with 1 ml cold PBS, resuspended with 1 ml PBS and centrifuged at 3 K for 2 min at 4 °C. Cells were resuspended in 1 ml PBS, 5 µl RNase A 10 µg/µl (Sigma) and 50 µg/ml propidium iodide (PI) (Sigma). PI-stained cells were filtered through a 40 µm mesh and incubated for 15–30 min in the dark. Cells were sorted by FACSARIA III (BD) based on their PI intensity to G1 and S phases^18^, using a flow rate of 1 and collected into 1.5 ml Protein-LoBind tubes (Eppendorf) and moved to ice.

### Whole-genome sequencing

DNA was extracted from cells using DNeasy-kit (QIAGEN) and eluted twice with 2 × (200 µl) of the kit elution buffer (AE), concentrated using 1.8 × Agencourt AMPure XP beads (Beckman Coulter) and eluted in 50 µl EB buffer (Qiagen). DNA concentrations were measured using Qubit dsDNA HS Assay Kit (Thermo Fisher Scientific). DNA samples were sonicated in the M220 Focused-Ultrasonicator (Covaris) using 50 W, 20% Duty Factor at 20 °C for 120 s, in order to reach an average target peak size of 250 bp, as verified with the D1000 or D1000 High Sensitivity ScreenTape, using the Electrophoresis 2200 TapeStation system (Agilent).

### Library preparation

Library preparation was carried out as described^45^. Briefly, sonicated DNA was subjected to a 50 µl end repair reaction using 1 µl End repair mix (NEB, #E6050L), cleaned by 1.8 × Ampure XP beads, followed by a 50-µl A-tail reaction, using 2 µl Klenow fragment exo-nuclease (NEB, #M0212L). Products were cleaned by 1.8 × beads and ligated by 2 µl quick ligase (NEB, #M2200) to 0.75 µM Illumina compatible forked indexed adapters. Ligation products were size selected by 0.7 × PEG (considering the PEG in the ligation buffer) in order to remove free adapters. 12–19 cycles of amplification were performed by PFU Ultra II Fusion DNA polymerase (Agilent, #600670) with the following Primers:

P7: 5’ AATGATACGGCGACCACCGAGATCTACACT CTTTCCCTACACGAC 3’; P5: 5’ CAAGCAGAAGACGGCATACGAGAT 3’. Amplified DNA was size selected for 300–700 bp fragments by taking the supernatant after using 0.5 × beads (which removed fragments greater than 700 bp), followed by a 1.0 × cleaning to remove remaining primers and adapter dimers. The final quality of the library was assessed by Qubit and TapeStation. Libraries were pooled and sequenced on NextSeq (Illumina) by 75 bp paired-end sequencing, generating ~10 M reads per library.

### Asynchronous replication-time assay

Sequenced reads were mapped using bowtie2 to the mm9 genome. Duplicates and reads with a mapping quality below 30 were removed. Clonal F1 reads were further analyzed to sort by allele as follows. We developed a two-step protocol for accurately assigning reads to the parental allele. First, we identified SNPs in each clone by mapping the reads using bowtie2 to the mm9 genome. SAMtools was used to create a pileup indicating the coverage of each base across the genome and BCFtools and the vcfutils.pl script were used to determine SNPs. Second, Reads were remapped using GSNAP^19^ with the predetermined SNP file in order for the Cast reads to be mapped permissibly to the mm9 genome. Only SNPs for which neither allele appeared less than 20% in the mapped reads, were used to separate overlapping reads to the appropriate genome. We removed reads (<7%) with contradictory SNP calls (more than 1 SNP, each belonging to a different strain), presumably generated by template switching during PCR amplification. Using this methodology, we avoided reference mapping bias, with the number of reads for each allele differing by less than 20%, on average. Reads for pure B6 cells were taken from previously published data^45^.

Determination of replication timing (RT) and calculation of differential RT regions were performed as described^18^. Briefly, G1 phase reads were binned and corresponding S-phase reads were counted in order to determine an S/G1 ratio for each bin. Ratio data was normalized, smoothed and interpolated using the Matlab csaps function (with a smoothing parameter of 10^−16^) at a resolution of 100 kb (approximate average size of the windows). Continuous segments containing less than 15 informative windows were removed from the analysis. Windows for which the ratio of B6/Cast G1 reads were more than 2 standard deviations away from the mean were removed. Differential regions were determined using the likelihood-ratio test at each genomic window as described^45^. The null model assumes that for each bin, all six RT measures come from the same distribution with a given mean, whereas the alternative model assumes that the replicates of each sample belong to two separate distributions, each with its own mean. FDR correction (Benjamini–Hochberg) was used to control for multiple testing. All regions with a q value below 0.01 were selected as differential and extended until the q value exceeded 0.05. Regions were further filtered to include only those that contain at least one window with a mean RT difference >0.5 between the two samples. The master list (Supplementary Table 1) was created by combining all asynchronous regions from each clone and then removing overlapping pure B6 and Cast differential regions. We validated this approach by showing that differences in RT between the active and inactive X chromosome could be easily detected in the same cells (Supplementary Fig. 5).

### Genomic analysis

Genomic data were obtained from the UCSC genome browser for the mm9 genome. LAD (GSM426758) and Hi-C (GSM1551643) data were obtained from the GEO platform. Genomic data were calculated and averaged over 100 kb-sized genomic windows using the BEDtools tools package. RNA-seq and ATAC-seq data^29^ was obtained in bam format. For Fig. 3, RNA-seq and ATAC-seq reads were counted and normalized over 100 kb windows. For Supplementary Fig. 3, differentially expressed genes were determined using the DESeq2 R package (adjusted *P*-value<0.1). GO analysis was performed on all autosomal UCSC genes overlapping the master list of asynchronous regions using the GOrilla gene ontology tool. All autosomal UCSC genes, except those appearing in pure differential regions, were submitted as background.

Single-cell allelic replication-timing data were taken from 40 CBA/MSM hybrid single-cell 7 day-differentiated ESC samples isolated from mid-S phase^23^. Data were obtained from the GEO database (GSE108556) where each single cell was analyzed at 400 kb intervals to determine whether each allele had replicated. The number of times either allele replicated earlier than the other in each cell was counted separately for the asynchronous and synchronous regions. The analysis was limited to mid-S autosomal replicating regions (mean replication-time between −0.5 and 0.5).

### FISH analysis

FISH analysis was performed as previously described^7,20^. Briefly, cells were pulse labeled for 1 h with BrdU and isolated nuclei then fixed with methanol/acetic acid (3:1). After denaturation and dehydration, slides were hybridized with labeled probes (Table 1) prepared with the nick translation DNA labeling system (ENZ-42910§§ or NICK TRANSLATION KIT (Abbott; 8-7J00-01) with either green dUTP 50 nM (Abbott, 8-2N-32) or orange dUTP 50 nM (Abbott, 8-2N-33). BrdU was detected by an anti-BrdU antibody (NeoMarkers) followed by rhodamine-labeled anti-mouse antibody (Jackson Laboratories). Replication-timing profiles (percent single/single, single/double, and double/double) were determined by counting 100–300 BrdU-positive nuclei for each sample^7,46^. All samples were mounted in VECTASHIELD (Vector) and mixed with DAPI and images were recorded using confocal microscopy (Nikon). In cases where coordination was detected parallel and antiparallel asynchronous replicating BACs were labeled using two different colors. Nuclei with both probes presenting the single-double pattern were counted^47^.

To identify strain-specific deletions, mapped reads from pure B6 and Cast DNA samples as well as clone E-5 and clone 2, F1 samples were binned and counted in 50 kb windows across the genome excluding blacklisted regions. Each sample’s counts were normalized to reads per million. For each sample a ratio was calculated by dividing the sample count by the average B6 count at each window. Any region of at least two continuous windows containing a ratio above 2 or below 1/2 were considered as potential copy number changes. Deletions were chosen by manually selecting regions from this list which also overlapped with known deletions from the Sanger project^16^.

### Reporting summary

Further information on research design is available in the Nature Research Reporting Summary linked to this article.

## Supplementary information

Supplementary Information

Reporting Summary

## Data Availability

All data (Figs. 1–4, Supplementary Figs. 1–5) have been deposited in the GEO public database: GSE130926. All remaining data are available from the authors upon reasonable request.

## Code availability

The underlying Python code to separate allelic reads is available upon request.
