# Supplementary material for: Chromosomal coordination and differential structure of asynchronous replicating regions
Source: Nat Commun. 2021 Feb 15;12:1035. doi: 10.1038/s41467-021-21348-4 (PMC7884787; doi:10.1038/s41467-021-21348-4)
Supplement: Supplementary file 1 — Supplementary Information [file 41467_2021_21348_MOESM1_ESM.pdf]

Supplementary Information

Chromosomal coordination and differential structure of asynchronous replicating regions

Blumenfeld et al.

a

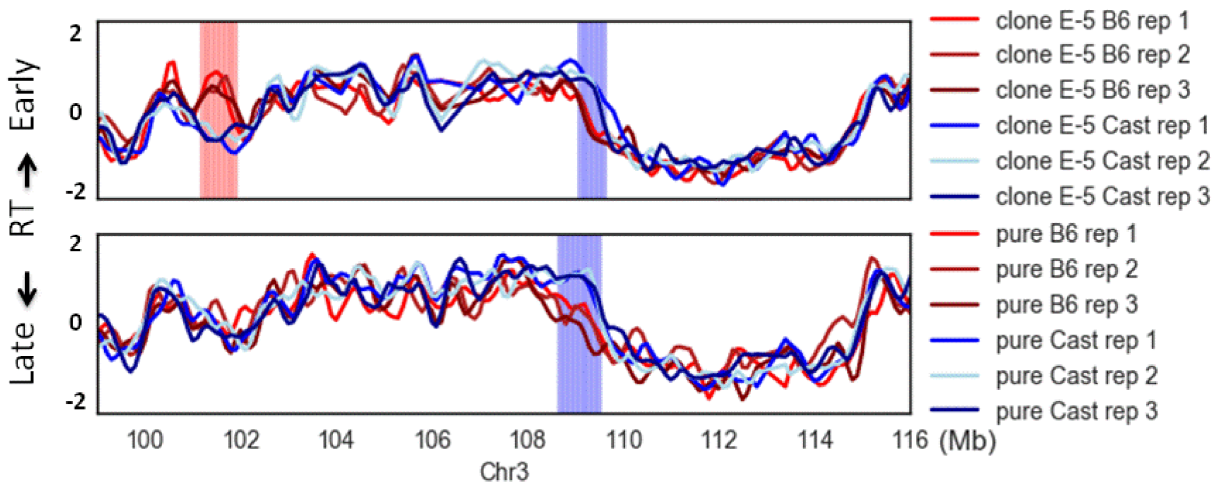

b

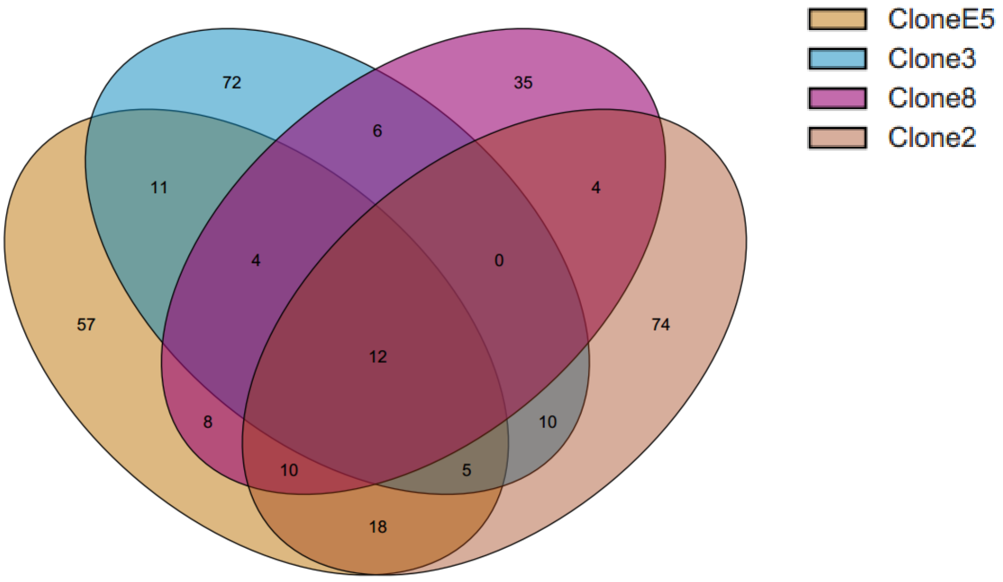

c

Clone name	# of AS regions	# of unique AS regions
Clone 2	133	133
Clone 3	114	93
Clone E-5	125	65
Clone 8	79	35

Supplementary Fig. 1. Genetic replication time differences between B6 and Cast. **(a)** Replication timing (RT) of the B6 (red) and Cast (blue) allele (three replicates) on Chr3 in clone E-5 (top) or a mixture of pre-B cells from pure B6 and Cast mice (bottom). Asynchronous regions detected in each experiment are marked by red (B6 early) or blue (Cast early) stripes. Note that one region (left) is asynchronously replicating exclusively in the clone, while the region on the right is genetically determined.
(b) Venn diagram showing the overlap of epigenetic autosomal AS-RT regions detected in different clones. **(c)** Table shows the number of total and unique asynchronous (AS) - RT regions identified in each pre-B cell clone. These AS-RT regions (n=326) range in size from 100 Kb to 1.7 Mb, with an average of 467 Kb.

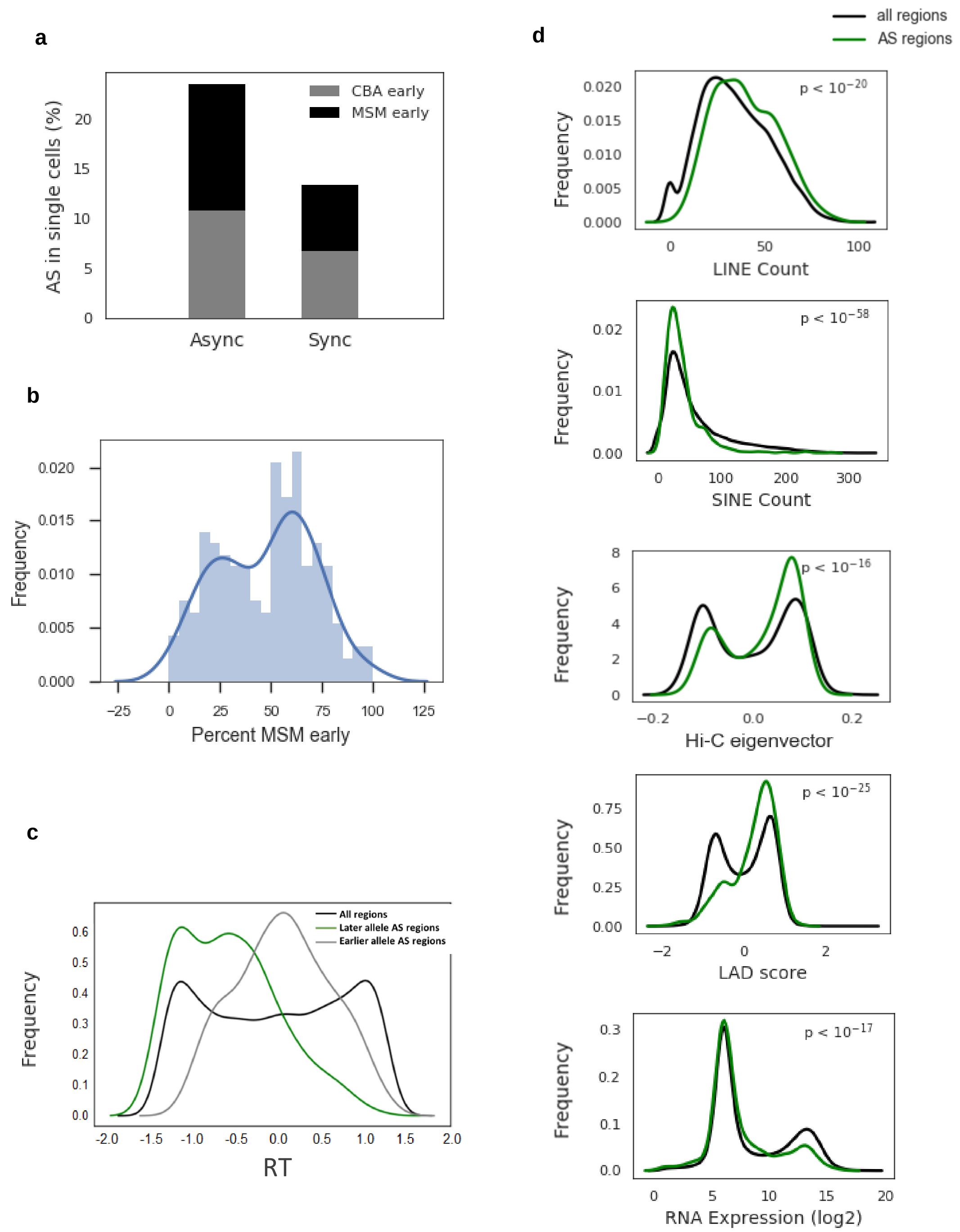

Supplementary Fig. 2. Characterization of AS replicating regions.
(a) Mid-S autosomal AS regions (Async) identified in our data (n=203) were scored for AS replication (see Materials and Methods) in a data base obtained from single CBA/MSM hybrid cell (n=40) analysis[1] that contained corresponding regions (n=186) and compared to the synchronous mid-S portion of the genome (Sync). The histogram also shows the proportion of regions showing early replication of either the CBA or MSM allele. P value < $5X10^{-5}$; two-tailed binomial test. **(b)** The percentage of cells where one allele replicated early and the other late was calculated for each window and the integrated frequency of MSM early replication was then graphed. Thus, most of these loci replicate the MSM allele early in some cells and late in others. The data was recorded in middle S phase. Synchronous regions show either both alleles (early) or none of the alleles (late). **(c)** Replication time (RT) distribution plot for the two alleles in AS-RT regions as compared to all regions in the genome. **(d)** Distribution plots comparing the AS regions, as derived from all pre-B cell clones, to the total genome for different characteristics, including LINE content, SINE content, the Hi-C eigenvector showing A and B compartments, LAD score, levels of RNA expression. P values were calculated using the two-sided Kolmogorov Smirnov test.

Supplementary Figure 3

a

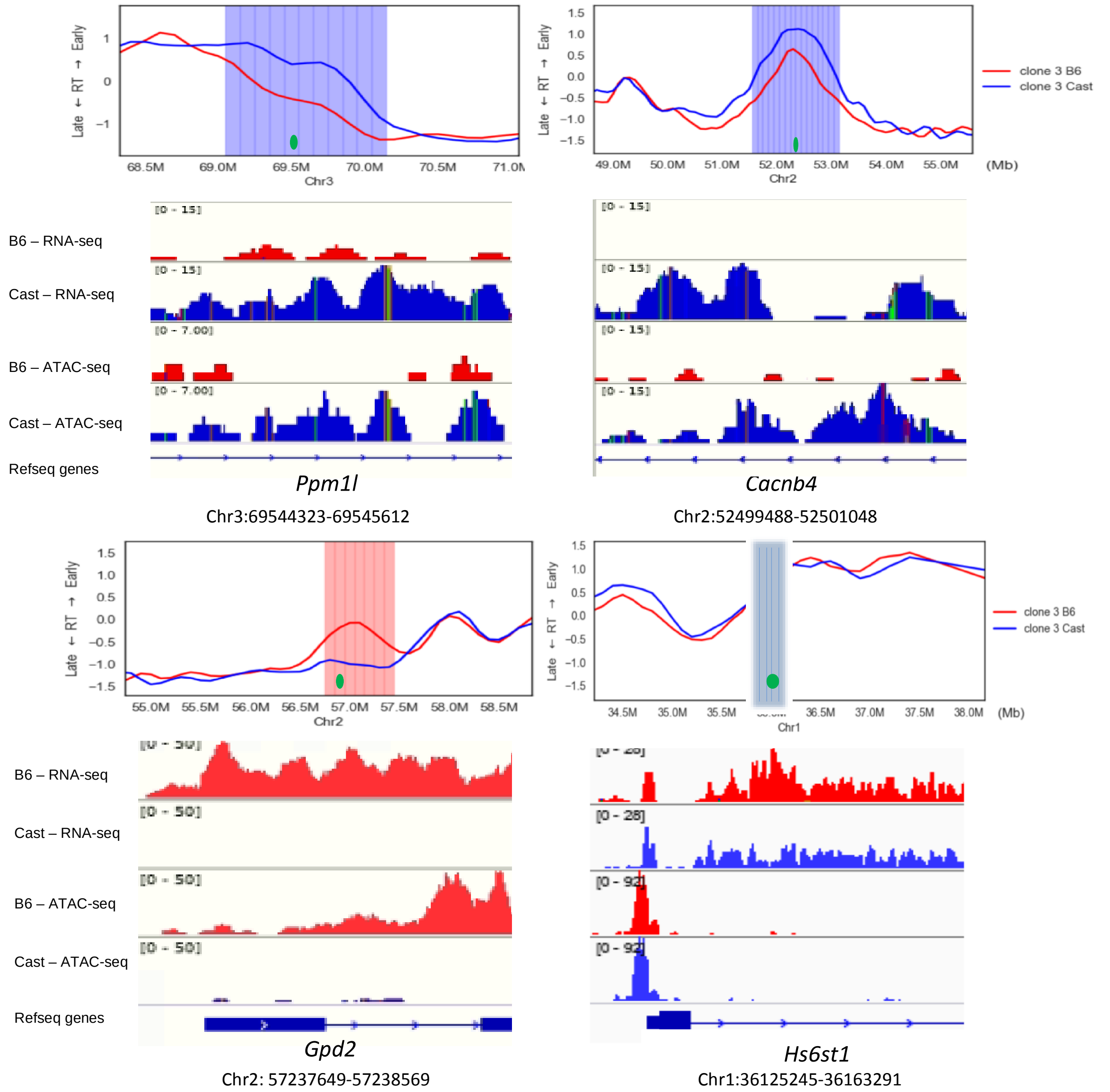

b

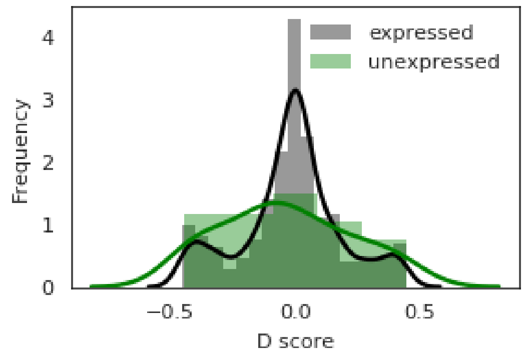

c

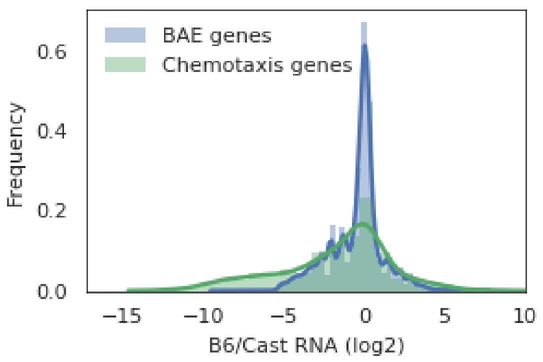

d

Go term	Genes
**Sensory perception of taste**	*Tas2r (103-107,109,110,113,114,117,119-125, 129,130,136,140)*
**G-protein coupled receptor signaling pathway**	*Hcrtr2, Rgs13*
**Cellular glucuronidation**	*Ugt1a1-Ugt1a10*
**Sensory perception of smell**	*Olfr (100, 103, 209, 498, 1205,1212,1240, 1271, 1263,1307,1506)*
**Neutrophil chemotaxis**	*Pde4d, Cxcl1,Cxcl3,Cxcl5,Cxcl12, Cxcl15, Pf4, Edn3, Itga1*
**Cellular ion homeostasis**	*Bcl2, Trdn, Drd1a, Slc9a1, Sc25a27*
**Cell motility**	*Enah, Cxcl3, Adamts12, Ric8, Pou3f3, Itga, fezf1*

Supplementary Fig. 3. Monoallelic expression analysis in asynchronously replicating regions.
(a) IGV track depicting gene-specific RNA-Seq coverage and ATAC profile for the B6 (red) and Cast (blue) alleles from **three** different asynchronously replicating regions in clone C3. The asynchronous replication timing (RT) pattern is shown above each example, with the green dots indicating the location of the gene itself. Gpd2 is in a B6 early region, while Ppm1l and Cacnb4 are located in Cast early-replicating regions, as determined by whole-genome analysis. Hs6st1(replicates synchronously) represents a typical example of genes expressed from both alleles.
(b) Distribution plot (relative frequency) of ATAC-Seq ratios (D score) for peaks located in AS regions (Supplementary Fig. 1) near genes which are either expressed (n=325, gray) or unexpressed (n=53, green) in these pre-B cells. The fact that the two distributions are similar (p = 0.15, two-sided Mann Whitney test), suggests that the differential ATAC-Seq pattern is independent of expression.
(c) Distribution of B6/Cast expression levels for the set of chemotaxis genes (green). Biallelic expressed (BAE) genes (blue) are shown as a control. **(d)** Table of GO terms and genes that are located in in AS-RT regions.

a

Cell type	Anti-parallel (%)
	A/B
E-5	81 (n=100)
C2	79 (n=105)
ESC	74 (n=61)

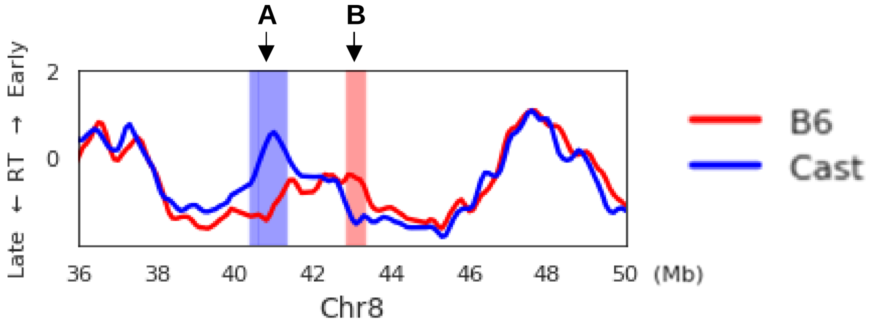

	A/B
E-5	75 (n=167)
C2	76 (n=146)
ESC	69 (n=102)

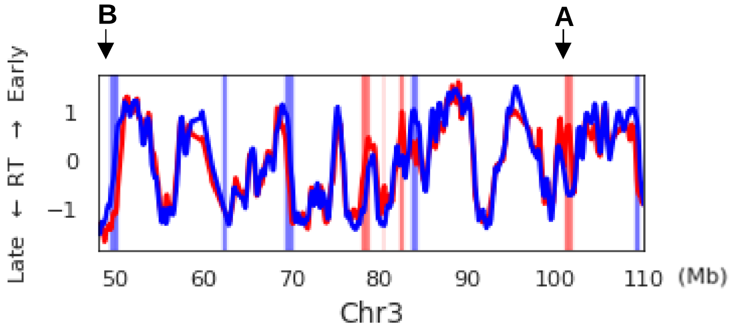

b

Cell type	Anti-parallel (%)	
	A/C	A/B
MEF	15 (n=63)	78 (n=68)
T-cells	15 (n=76)	79 (n=53)

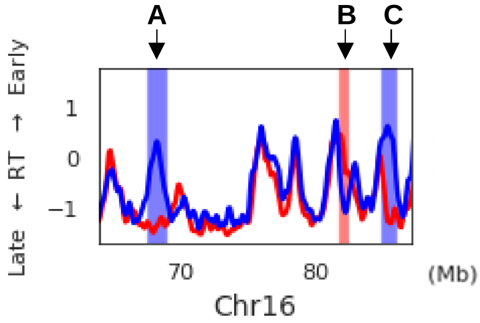

	A/C	A/B
MEF	16 (n=59)	82 (n=85)
T-cells	18 (n=100)	79 (n=72)

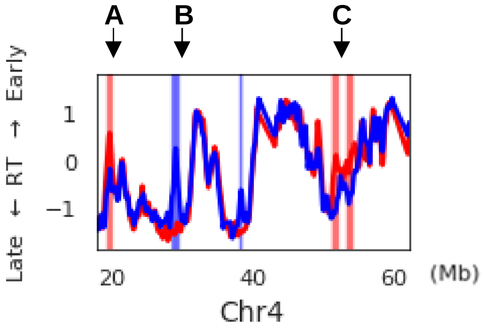

	A/C	A/B
MEF	13 (n=95)	77 (n=88)
T-cells	17 (n=91)	80 (n=78)

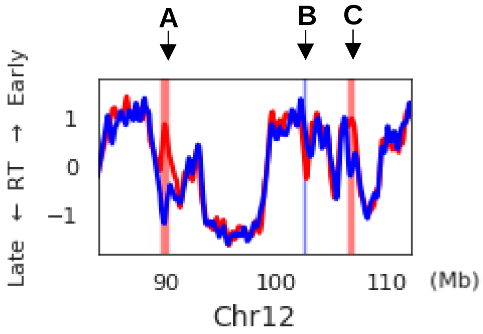

c

	Anti-parallel (%)
**chr21**	**RP11-790H8/RP11-701L12**
YC	73 (n=102)

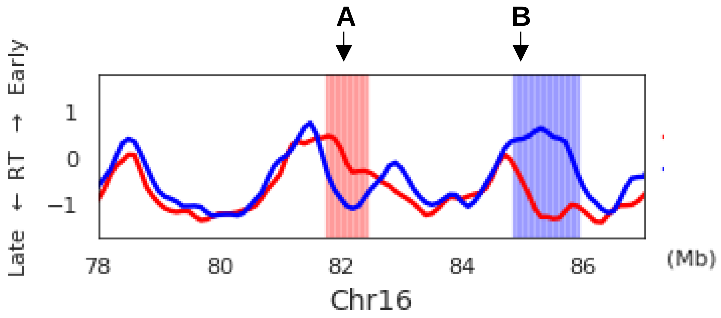

Supplementary Fig. 4. Chromosome coordination of asynchronous replication regions by FISH.

Double-label FISH was carried out in various cell populations using BAC probe pairs (A/B or A/C) for two separate asynchronously-replicating regions on a given chromosome (8, 3, 16, 4, 12) as determined by whole genome analysis in clone E-5 (map). Nuclei showing a single/double signal for both probes were selected and scored for percent having an anti-parallel pattern with each allele carrying a single signal for one probe together with a double signal for the other or a parallel pattern with each allele carrying a single signal or double signal for both probes. Coordination is shown for either two **(a)** or three **(b)** probes in mouse cells and two probes in human cells **(c)**. The maps show replication timing (RT) for the B6 and Cast alleles (average of three replicates). The maximum p value for all cases was $< 2X10^{-4}$ as determined by the two-tailed binomial test. Probes: mouse Chr8 (A=RP24-318L18, B=RP23-182L17); Mouse Chr3 (A=RP23-115D3, B=RP23-317D10); mouse Chr16 A=RP23-38D22, B=RP23-326K16, C=RP23-59L11); mouse Chr4 (A=RP24-83L7, B=RP23-439A9, C=RP23-63H2); mouse Chr12 (A=RP23-13F5, B=RP24-386G17, C=RP23-333P23), human Chr21 (A=RP11-790H8, B=RP11-701L12), which is syntenic to the AS region on mouse Chr16.

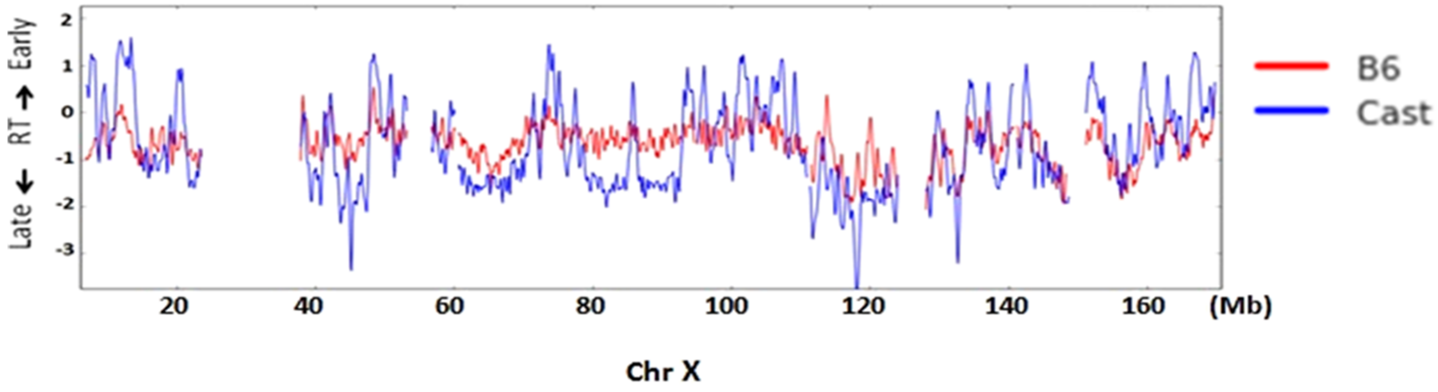

Supplementary Fig. 5. X-chromosome allele-specific replication timing. The active X chromosome (Cast, blue) has a normal replication timing (RT) profile while the inactive X (B6, red) shows a relatively constant RT profile over the entire length. These results are in agreement with published data[2,3].

Supplementary Table 1

Chr #	Start *	End *	Early allele	Chr #	Start *	End *	Early allele	Chr #	Start *	End *	Early allele
chr1	12450000	12850000	CAST	chr2	112950000	113150000	C57BL	chr4	66250000	67050000	C57BL
chr1	24150000	24450000	CAST	chr2	113150000	113350000	CAST	chr4	71050000	71750000	CAST
chr1	32750000	33350000	CAST	chr2	118050000	118150000	CAST	chr4	74250000	75750000	CAST
chr1	40050000	41350000	CAST	chr2	119150000	119550000	CAST	chr4	78550000	79450000	CAST
chr1	42250000	42850000	CAST	chr2	121150000	121250000	CAST	chr4	84150000	84750000	C57BL
chr1	45450000	45650000	CAST	chr2	121250000	121550000	CAST	chr4	104850000	105150000	CAST
chr1	46850000	47550000	C57BL	chr2	127650000	128350000	CAST	chr4	111250000	112350000	C57BL
chr1	56750000	56950000	CAST	chr2	130250000	130550000	CAST	chr4	112750000	113550000	CAST
chr1	73450000	73650000	CAST	chr2	146350000	146550000	CAST	chr4	113750000	113850000	CAST
chr1	77150000	77350000	CAST	chr2	149050000	149350000	C57BL	chr4	113950000	114050000	CAST
chr1	80050000	80650000	C57BL	chr2	172550000	172650000	CAST	chr4	129150000	129450000	CAST
chr1	84550000	84950000	C57BL	chr2	174250000	174750000	C57BL	chr4	132750000	133150000	CAST
chr1	89950000	90250000	C57BL	chr3	4650000	4950000	C57BL	chr4	141750000	141850000	CAST
chr1	100150000	100250000	CAST	chr3	19450000	19750000	CAST	chr5	12350000	13350000	C57BL
chr1	100250000	100650000	CAST	chr3	27450000	27950000	CAST	chr5	21450000	22050000	C57BL
chr1	107850000	108950000	C57BL	chr3	40850000	41150000	CAST	chr5	26450000	26550000	CAST
chr1	122450000	122550000	C57BL	chr3	49450000	50350000	CAST	chr5	28950000	29750000	CAST
chr1	128150000	128850000	CAST	chr3	61950000	62050000	CAST	chr5	59750000	61450000	C57BL
chr1	134350000	134650000	CAST	chr3	62250000	62650000	CAST	chr5	68750000	69450000	C57BL
chr1	142650000	143150000	C57BL	chr3	63550000	63750000	CAST	chr5	77150000	77550000	CAST
chr1	145950000	146350000	C57BL	chr3	66850000	67950000	C57BL	chr5	77850000	78050000	CAST
chr1	160050000	161150000	C57BL	chr3	69050000	69150000	CAST	chr5	80850000	81050000	C57BL
chr1	163150000	163250000	CAST	chr3	69350000	70250000	CAST	chr5	81250000	82350000	C57BL
chr1	163450000	163550000	CAST	chr3	74650000	75750000	CAST	chr5	84150000	84250000	C57BL
chr1	163950000	164450000	CAST	chr3	77950000	78050000	C57BL	chr5	91050000	91550000	CAST
chr1	183750000	184250000	C57BL	chr3	78050000	78850000	C57BL	chr5	96950000	98350000	C57BL
chr1	187850000	188650000	C57BL	chr3	80450000	80650000	C57BL	chr5	102050000	102550000	C57BL
chr1	190650000	191150000	CAST	chr3	82350000	82750000	C57BL	chr5	105450000	105850000	CAST
chr1	191450000	192050000	C57BL	chr3	83350000	83450000	C57BL	chr5	109650000	109950000	CAST
chr2	15650000	17050000	CAST	chr3	83650000	84350000	CAST	chr5	130550000	130850000	C57BL
chr2	23850000	24150000	C57BL	chr3	87150000	87250000	CAST	chr5	130950000	131350000	C57BL
chr2	44450000	44750000	CAST	chr3	100950000	101050000	C57BL	chr5	146950000	147250000	C57BL
chr2	50650000	51250000	CAST	chr3	101150000	101950000	C57BL	chr5	147350000	147650000	C57BL
chr2	51550000	53150000	CAST	chr3	104050000	104250000	CAST	chr6	22550000	23750000	CAST
chr2	56750000	57450000	C57BL	chr3	122850000	122950000	CAST	chr6	33950000	34350000	CAST
chr2	64850000	65550000	C57BL	chr3	123450000	123850000	C57BL	chr6	35050000	35250000	C57BL
chr2	65850000	67450000	CAST	chr3	124250000	124550000	C57BL	chr6	46650000	47050000	CAST
chr2	68050000	68950000	CAST	chr3	126850000	126950000	CAST	chr6	50650000	51150000	C57BL
chr2	71550000	72050000	CAST	chr4	5250000	5850000	CAST	chr6	59150000	59750000	CAST
chr2	75050000	75350000	C57BL	chr4	12750000	13450000	CAST	chr6	66550000	67450000	CAST
chr2	76150000	76550000	C57BL	chr4	19550000	20250000	C57BL	chr6	69850000	70050000	C57BL
chr2	76750000	77250000	C57BL	chr4	21350000	21650000	C57BL	chr6	70150000	70550000	C57BL
chr2	81150000	81350000	CAST	chr4	28650000	29750000	CAST	chr6	73350000	73650000	CAST
chr2	83650000	83750000	CAST	chr4	31950000	32050000	CAST	chr6	73650000	73950000	CAST
chr2	88650000	90150000	C57BL	chr4	38250000	38650000	CAST	chr6	84250000	84450000	CAST
chr2	97450000	97950000	C57BL	chr4	51250000	52150000	C57BL	chr6	85450000	85550000	CAST
chr2	111750000	111850000	C57BL	chr4	53250000	54250000	C57BL	chr6	85750000	85950000	CAST
chr2	112350000	112450000	C57BL	chr4	54650000	54950000	C57BL	chr6	93050000	94350000	CAST
chr2	112550000	112850000	C57BL	chr4	63350000	63950000	CAST	chr6	106450000	106550000	CAST

Chr #	Start *	End *	Early allele	Chr #	Start *	End *	Early allele	Chr #	Start *	End *	Early allele
chr6	111950000	112450000	CAST	chr9	41850000	42050000	C57BL	chr12	106450000	107050000	C57BL
chr6	131350000	131450000	CAST	chr9	53650000	53950000	CAST	chr12	116550000	116850000	CAST
chr6	131550000	131750000	CAST	chr9	61950000	62450000	C57BL	chr13	3550000	4150000	C57BL
chr6	131850000	132450000	CAST	chr9	75550000	76250000	CAST	chr13	5250000	5850000	CAST
chr6	132550000	133050000	CAST	chr9	79550000	80050000	C57BL	chr13	6550000	7350000	C57BL
chr6	137050000	137250000	CAST	chr9	94750000	95050000	C57BL	chr13	11450000	11550000	CAST
chr6	139150000	139250000	CAST	chr9	101850000	102050000	CAST	chr13	22850000	23450000	CAST
chr7	17750000	18150000	CAST	chr9	102150000	102550000	CAST	chr13	42950000	43450000	C57BL
chr7	30950000	31250000	CAST	chr9	104650000	104850000	CAST	chr13	51350000	51650000	C57BL
chr7	32150000	32950000	CAST	chr9	105450000	105750000	CAST	chr13	54050000	54150000	CAST
chr7	37250000	37350000	CAST	chr9	105850000	106150000	CAST	chr13	56650000	58150000	CAST
chr7	37450000	37550000	CAST	chr9	111850000	112650000	C57BL	chr13	64550000	64750000	CAST
chr7	54450000	54550000	C57BL	chr9	115250000	115650000	CAST	chr13	66250000	67050000	CAST
chr7	54750000	55050000	C57BL	chr9	117050000	117150000	C57BL	chr13	73650000	74350000	C57BL
chr7	57550000	57950000	CAST	chr10	9650000	10150000	C57BL	chr13	89950000	90550000	CAST
chr7	72150000	72450000	C57BL	chr10	11550000	12150000	CAST	chr13	102150000	103150000	C57BL
chr7	73850000	74250000	CAST	chr10	21550000	21950000	CAST	chr13	110350000	110750000	C57BL
chr7	75150000	75750000	C57BL	chr10	27850000	28550000	CAST	chr13	115850000	116250000	CAST
chr7	89450000	89950000	CAST	chr10	32650000	32750000	C57BL	chr14	9450000	9850000	CAST
chr7	97350000	97950000	CAST	chr10	32950000	33150000	C57BL	chr14	11250000	11550000	CAST
chr7	115550000	115650000	CAST	chr10	34050000	34350000	C57BL	chr14	18150000	19050000	C57BL
chr7	116050000	116650000	CAST	chr10	43250000	43350000	C57BL	chr14	23950000	24750000	CAST
chr7	120150000	120450000	C57BL	chr10	51650000	52050000	CAST	chr14	45450000	45550000	CAST
chr7	121750000	122050000	CAST	chr10	53750000	53850000	C57BL	chr14	53550000	54450000	CAST
chr7	122850000	123150000	C57BL	chr11	13750000	14350000	C57BL	chr14	71050000	71150000	CAST
chr7	123950000	124350000	CAST	chr11	15750000	16950000	C57BL	chr14	86150000	87150000	C57BL
chr7	130650000	131350000	CAST	chr11	27250000	27650000	C57BL	chr14	89450000	89550000	CAST
chr7	131650000	132150000	CAST	chr11	28150000	28950000	CAST	chr15	11050000	11150000	C57BL
chr7	136150000	136750000	CAST	chr11	29850000	30550000	C57BL	chr15	13250000	13450000	C57BL
chr7	137050000	137150000	C57BL	chr11	52550000	52650000	C57BL	chr15	22150000	23050000	CAST
chr7	147850000	148150000	C57BL	chr11	56850000	57350000	CAST	chr15	24950000	25750000	CAST
chr7	152150000	152350000	CAST	chr11	58750000	58950000	CAST	chr15	30350000	30750000	C57BL
chr8	24450000	25150000	CAST	chr11	64950000	65350000	CAST	chr15	31350000	31750000	C57BL
chr8	25250000	25650000	CAST	chr11	81150000	81650000	CAST	chr15	31850000	32550000	C57BL
chr8	29250000	29550000	CAST	chr12	4550000	4750000	CAST	chr15	32550000	32650000	C57BL
chr8	31550000	31850000	CAST	chr12	7850000	8550000	CAST	chr15	34150000	34250000	CAST
chr8	32050000	32350000	CAST	chr12	15350000	15750000	CAST	chr15	34550000	34650000	CAST
chr8	35450000	35950000	CAST	chr12	28450000	28650000	CAST	chr16	7350000	7450000	CAST
chr8	37550000	37650000	CAST	chr12	29750000	30450000	C57BL	chr16	8950000	9850000	CAST
chr8	40350000	41350000	CAST	chr12	30650000	31250000	CAST	chr16	19350000	19650000	C57BL
chr8	42850000	43350000	C57BL	chr12	34950000	35850000	C57BL	chr16	37950000	38050000	CAST
chr8	64050000	65050000	CAST	chr12	45750000	46850000	C57BL	chr16	38850000	39150000	CAST
chr8	69950000	70150000	C57BL	chr12	53550000	54350000	C57BL	chr16	40250000	40650000	CAST
chr8	71150000	71250000	CAST	chr12	58950000	59450000	CAST	chr16	45350000	45750000	CAST
chr9	9050000	9450000	C57BL	chr12	60250000	60550000	CAST	chr16	48250000	49050000	C57BL
chr9	23850000	24150000	CAST	chr12	76250000	76450000	C57BL	chr16	51750000	51850000	CAST
chr9	28550000	29450000	C57BL	chr12	89650000	90450000	C57BL	chr16	59150000	59650000	C57BL
chr9	31050000	31350000	C57BL	chr12	102450000	102750000	CAST	chr16	66650000	66750000	CAST
chr9	41250000	41350000	CAST	chr12	105150000	105250000	CAST	chr16	67650000	69050000	CAST

Chr #	Start *	End *	Early allele
chr16	69650000	69950000	C57BL
chr16	81750000	82450000	C57BL
chr16	82450000	83250000	CAST
chr16	84850000	85950000	CAST
chr16	87150000	87350000	CAST
chr16	87650000	88050000	CAST
chr16	89250000	90250000	CAST
chr17	4250000	4650000	C57BL
chr17	10350000	10950000	C57BL
chr17	19050000	19250000	CAST
chr17	24150000	24350000	CAST
chr17	35150000	35250000	CAST
chr17	35650000	35850000	CAST
chr17	36650000	36950000	C57BL
chr17	37450000	37650000	CAST
chr17	43350000	44250000	CAST
chr17	69450000	69850000	CAST
chr17	69950000	70050000	CAST
chr17	70150000	70950000	CAST
chr17	72450000	73050000	CAST
chr18	9650000	9950000	CAST
chr18	15550000	15950000	CAST
chr18	21750000	22150000	C57BL
chr18	22850000	23450000	C57BL
chr18	24350000	24450000	C57BL
chr18	28050000	29050000	C57BL
chr18	32850000	33650000	CAST
chr18	41650000	41750000	CAST
chr18	47150000	47550000	CAST
chr18	47850000	48150000	CAST
chr18	52850000	53150000	CAST
chr18	54850000	55350000	C57BL
chr18	58250000	59150000	CAST
chr18	76250000	76450000	CAST
chr18	78250000	78950000	C57BL
chr19	3550000	3650000	CAST
chr19	3850000	3950000	C57BL
chrX	7050000	8050000	CAST
chrX	8750000	8850000	CAST
chrX	8850000	9150000	CAST
chrX	9250000	9350000	CAST
chrX	11050000	13350000	CAST
chrX	13450000	13750000	CAST
chrX	15450000	15850000	C57BL
chrX	19450000	20550000	CAST
chrX	21150000	22850000	C57BL
chrX	36250000	37050000	C57BL
chrX	40150000	41950000	C57BL
chrX	43350000	44450000	C57BL
chrX	45250000	46750000	CAST
chrX	48050000	48350000	CAST
chrX	50450000	50650000	CAST
chrX	55250000	55650000	C57BL
chrX	55950000	56450000	C57BL
chrX	56750000	56950000	CAST
chrX	57050000	57150000	CAST
chrX	58150000	65750000	C57BL
chrX	66250000	67450000	C57BL
chrX	70850000	71650000	CAST
chrX	72350000	72850000	CAST
chrX	72950000	73450000	C57BL
chrX	73950000	74250000	C57BL
chrX	74450000	74550000	CAST
chrX	74950000	75050000	CAST
chrX	76150000	82450000	C57BL
chrX	82850000	83350000	CAST
chrX	83550000	89950000	C57BL
chrX	90250000	90350000	CAST
chrX	90750000	91250000	CAST
chrX	92950000	93250000	CAST
chrX	95050000	95450000	CAST
chrX	96850000	97450000	C57BL
chrX	98050000	99550000	CAST
chrX	101550000	101850000	CAST
chrX	103250000	103350000	CAST
chrX	103950000	104750000	CAST
chrX	105950000	106550000	CAST
chrX	107950000	108050000	C57BL
chrX	108550000	108750000	C57BL
chrX	109350000	111050000	C57BL
chrX	111250000	118450000	C57BL
chrX	118650000	119950000	C57BL
chrX	124750000	125450000	C57BL
chrX	128250000	129050000	C57BL
chrX	131250000	131750000	CAST
chrX	133450000	133850000	CAST
chrX	134050000	136050000	C57BL
chrX	136050000	136250000	C57BL
chrX	136650000	137350000	CAST
chrX	138550000	139150000	CAST
chrX	140350000	146050000	C57BL
chrX	146550000	146850000	C57BL
chrX	147550000	147650000	CAST
chrX	147750000	147950000	CAST
chrX	148050000	148550000	CAST
chrX	148950000	149050000	CAST
chrX	149350000	149550000	CAST
chrX	149750000	150350000	CAST
chrX	152850000	153250000	C57BL
chrX	155750000	156250000	CAST
chrX	157650000	158150000	C57BL
chrX	159250000	159650000	CAST
chrX	162350000	162650000	CAST
chrX	162650000	163950000	CAST
chrX	165450000	165750000	CAST

Supplementary references

1. Takahashi, S. *et al.* Genome-wide stability of the DNA replication program in single mammalian cells. *Nat. Genet.* **51,** 529–540 (2019).
2. Koren, A. & McCarroll, S. A. Random replication of the inactive X chromosome. *Genome Res.* **24,** 64–69 (2014).
3. Casas-Delucchi, C. S. *et al.* Histone acetylation controls the inactive X chromosome replication dynamics. *Nat. Commun.* **2,** 222 (2011).
